# 外泌体提取方法及在肺癌早期诊断中的作用研究进展

**DOI:** 10.3779/j.issn.1009-3419.2020.101.24

**Published:** 2020-11-20

**Authors:** 丹 罗, 春雷 李, 伦 武, 琴华 陈

**Affiliations:** 1 442008 十堰，湖北医药学院附属东风总医院医学实验中心，湖北医药学院 Department of Medical Experimental Center, Affiliated Dongfeng Hospital, Hubei University of Medicine, Shiyan 442008, China; 2 442000 十堰，湖北医药学院药学院 Hubei University of Medicine, College of Pharmacy, Shiyan 442000, China; 3 518102 深圳，深圳市宝安纯中医治疗医院 Shenzhen Baoan Authentic Traditional Chinese Medicine Therapy Hospital, Shenzhen 518102, China

**Keywords:** 外泌体, 肺肿瘤, 早期诊断, Exosomes, Lung neoplasms, Early diagnosis

## Abstract

肺癌是世界范围内发病率和死亡率较高的恶性肿瘤之一，严重威胁着国民的生命安全与健康。肺癌的早期诊断是肺癌预防和治疗过程中的关键环节，对肺癌进行早期诊断有利于提高患者的生存率。外泌体(exosomes)与肿瘤的侵袭与转移过程密切相关，在肺癌的发生发展过程中，外泌体发挥着重要的调控作用。近年来，以外泌体为载体的生物标记物成为肺癌强有力的诊断工具。外泌体是一种由细胞分泌的由膜包裹的大小均一、直径约为30 nm-200 nm的脂质双分子层结构小囊泡。外泌体的内容物包含不同类型的核酸和蛋白质，这些核酸和蛋白质来源于其亲本细胞(包括亲本癌细胞)，具有广泛的生理功能，包括参与免疫调节、细胞间联络等。外泌体中的生物大分子物质，如单链RNA、长非编码RNA、微小RNA(microRNA, miRNA)、蛋白质以及脂类，可以为肺癌的早期临床诊断提供有价值的信息。因此，本文就外泌体的来源、结构特点、提取方法、生物学特性和在肺癌早期诊断中的作用研究进展做简要阐述。

## Abstract (en)

Lung cancer is a malignant tumor with high incidence and mortality in the world, which seriously threatens people's safety and health. Early diagnosis of lung cancer is the key part in the process of prevention and treatment of lung cancer. It can improve the survival of patients with lung cancer. Exosomes are closely related to the invasion and metastasis process of tumor, it plays an important role in the development of lung cancer. Biomarkers based on exosomes have become a powerful diagnostic tool of lung cancer. Exosomes are lipid bilayer vesicles with uniform size and diameter of 30 nm-200 nm secreted by cells. Exosomes contain different types of nucleic acids and proteins. These nucleic acids and proteins are derived from their parent cells (including parent cancer cells), which have a wide range of physiological functions, including immune regulation, intercellular communication and other physiological activities. Biomacromolecules in exosomes, such as single-stranded RNA, long noncoding RNA, microRNA, protein and lipids, which can provide valuable genetic information for early clinical diagnosis of lung cancer. This review described the origin, structural characteristics, extraction methods, biological characteristics of exosomes and the relationships of exosomes in the early diagnosis of lung cancer.

肺癌是世界范围内发病率较高的恶性肿瘤之一^[1]^，它的死亡率在各类癌症中高居榜首，并明显呈现出逐年上升的趋势^[2]^，是一种严重威胁国民生命健康的重大疾病。目前，由于缺乏精准有效的早期诊断方法、肿瘤细胞容易转移和治疗后复发率较高等各种原因，肺癌患者5年生存率较低，总体上不超过15%^[3]^。尽管目前国内外有很多的药物研发、肺癌发生发展研究和为改进肺癌治疗策略所做的努力，但肺癌仍然是预后不良的一种典型疾病^[4]^。

肺癌的早期诊断是肺癌预防和治疗过程中的关键环节之一，但目前可以用于肺癌早期诊断的方法非常局限，胸部X线敏感性较差^[5]^、螺旋计算机断层扫描(computed tomography, CT)敏感性高，但准确性和特异性较差^[6]^。肺癌的早期诊断依然面临着巨大的技术瓶颈和挑战，大多数肿瘤体积已经生长到足够大时甚至肿瘤细胞已经开始转移时才能够被传统的诊断技术检测出来^[7]^，这时患者病情已经处于中晚期，进行针对性治疗比较困难并且预后情况较差，这导致肺癌患者的总体生存率较低^[8]^。目前在肺癌的临床诊断和治疗中所采用的肿瘤原发灶-淋巴结-转移(tumor-node-metastasis, TNM)分期标准具有准确性较差、分型效率较低、不能有效地指导临床治疗和预后判断等缺点^[9]^，尤其是对于开展肺癌患者的个体化治疗及预后评估等方面所发挥的作用极为有限。早发现、早诊断、早治疗对于降低肺癌患者的死亡率，提高其生存率具有极其重要的意义，而外泌体是肺癌早期诊断中极具前景的一类检测对象，在肺癌的发生发展过程中发挥着重要作用。外泌体内部含有丰富的生物大分子物质，可以为肺癌的早期临床诊断提供有价值的信息，这在肺癌早期诊断中具有重要意义。

## 1 液体活检在肺癌早期诊断中的意义

“液体活检”技术是一种非侵入式的微创的疾病检测方法，基于液体活检的早期分子诊断技术^[10]^，近年来逐渐成为一个研究热点。它区别于传统的手术活检技术和穿刺活检技术，主要利用癌症患者的体液如血液、尿液、乳汁、唾液等来检测肿瘤的循环生物标志物，从而获得相关疾病的遗传信息^[11]^，为疾病的早期诊断和治疗提供新的思路和方法。液体活检具有操作简便、非侵入式^[12, 13]^、成本较低、副作用小^[14]^、样品采集方便、可以重复取样、对于患者的伤害较小、患者的可接受程度较高等^[15]^优势，可早于影像学条件下发现肿瘤，适合用于相关疾病的早期诊断。这也为肺癌患者的早期诊断和辅助临床治疗提供了一种新思路和新途径，从而改善肺癌患者预后情况、提高肺癌患者的生活质量并且降低肺癌患者的死亡率。

大量研究表明，通过检测和分析肿瘤患者外周循环血液中存在的生物标志物，如循环肿瘤细胞(circulating tumor cells, CTCs)^[16]^、外泌体、DNA或RNA等物质，可以探知肿瘤相关的基因信息，这也为检测和监控肿瘤的动态演变提供了一个强有力的证据^[17]^。CTCs是从实体肿瘤中脱离出来并进入外周血液循环的肿瘤细胞。CTCs的数目一般较少，而血液中血细胞的数量庞大，检测CTCs的关键在于根据CTCs的物理学特征(大小、密度、电荷、可变形性)和生物学特征(表面抗原、侵袭性等)，来实现CTCs从血液中的识别和富集^[18]^。目前不管是通过物理学特征和生物学特征来识别CTCs都面临着巨大的技术挑战，CTCs的异质性对识别和富集方法会产生一定的影响，且肿瘤患者血液中为数不多的CTCs很难完整反映整个肿瘤的整体信息。而由肿瘤细胞释放到各种体液中的外泌体含量丰富，这些外泌体与肿瘤的发生、发展以及转移具有一定的相关性。因此，利用肿瘤细胞释放到血液中的外泌体，检测其中特异性的遗传信息等，可以分析肿瘤相关的信息。通过液体活检技术连续监测肺癌患者外周循环血液中肿瘤标志物的变化，可进一步了解肺癌的发生发展过程^[19]^。因此，将液体活检技术广泛应用于肺癌早期诊断的工作中，是目前迫切亟需解决的问题，也可为肺癌早期诊断的临床研究奠定坚实的基础。

## 2 外泌体在液体活检早期诊断中的作用和意义

在肺癌液体活检的各种检测物质中，最具有检测前景的一类检测对象是肺癌来源的外泌体。外泌体最初被Johnstone等描述为一种由网织红细胞分泌的独特的物质小泡，1987年“外泌体”一词被提出用来指代那些在细胞内形成、释放于细胞外的微小型细胞外囊泡^[20]^。近年来多项研究表明，由于外泌体存在于人体的大多数体液中，其稳定性比较好并且其内容物与亲本细胞具有较大的相似性^[21]^，通过检测和提取外泌体中的内容物可以获知其亲本细胞相关的遗传物质和信息。人体各种体液中的外泌体含量丰富、特异性高、大小均匀，包含丰富的同肿瘤相关的基因信息，如特异性生物标志物(RNAs、DNAs、脂质和蛋白质)等生物分子，直接或间接调控受体细胞的表达，在肿瘤的发生发展过程中起着极其重要的调控作用。目前已有一系列的临床研究利用外泌体作为生物标志物，Cecilia等^[22]^研究发现miR-21的表达增加与非小细胞肺癌(non-small cell lung cancer, NSCLC)患者的预后恶化有关。Zhou等^[23]^筛选了肺癌患者外泌体中的6种miRNA，包括miR-19-3p、miR-21-5p、miR-221-3p、miR-409-3p、miR-425-5p和miR-584-Sp，其中miR-19-3p、miR-21-5p和miR-221-3p与正常人相比表达上调，差异具有统计学意义。通过精确检测分析外泌体内容物的类型、含量等特性，可以辅助肺癌的早期诊断。因此，外泌体具有很大的潜力发展成为一种肺癌早期诊断的生物标志物，作为肺癌的液体活检工具^[24]^，对肺癌的早期诊断具有重要价值。

### 2.1 外泌体概述

外泌体是一种由细胞主动分泌的由膜包裹的^[25]^大小均一、直径约为30 nm-200 nm的脂质双分子层结构小囊泡^[26]^。外泌体的内容物包含不同类型的核苷酸和蛋白质，这些核苷酸和蛋白质来源于其亲本细胞(包括亲本癌细胞)^[27]^，外泌体广泛分布于各种体液中，如血液、尿液、乳汁和唾液等^[28]^。外泌体是在质膜双内陷和细胞内多泡小体(multivesicular bodies, MVBs)形成过程中产生的，外泌体起源于质膜循环途径中的膜腔或早期胞内体^[29]^，它们向内凹陷形成管腔内膜泡，逐渐发展成为MVBs，MVBs在细胞内发生移动与其亲本细胞质膜表面进行融合，然后通过胞吐作用以外泌体的形式分泌至细胞外，最终形成外泌体^[30]^。血浆膜的第一次内陷形成一个杯状结构，包括细胞表面蛋白和与胞外环境相关的可溶性蛋白。MVBs既可以与溶酶体或自噬体融合降解，也可以与质膜融合释放含有腔内小泡的外泌体^[30]^。外泌体的组成成分繁多而复杂，其内部含有丰富的生物学大分子物质，如单链RNA、长链非编码RNA(long non-coding RNA, lncRNA)、微小RNA(microRNA, miRNA)、蛋白质、脂类^[31-33]^、氨基酸和代谢物，外泌体的内容物可以反映它们的起源细胞，可为肿瘤的临床诊断方面提供有价值的遗传信息。

### 2.2 外泌体生物学作用

外泌体最早被认为是细胞内信息交流的一种运输载体，此后，有研究者^[24]^发现外泌体具有免疫调控和抗原呈递作用。外泌体可介导细胞与细胞间的相互交流，这与其含有大量的核苷酸(mRNA、miRNA、lncRNA、核糖体RNA等)、蛋白质(膜转运蛋白、融合蛋白、热休克蛋白等)、脂质(胆固醇、磷脂、甘油二脂等)密切相关^[34]^。其中，CD63、CD81等蛋白是外泌体特有的标志性蛋白^[35]^，可作为外泌体的生物学鉴定标志。各细胞间信息交流的异常调节和异常交换导致了肿瘤的发生。在肿瘤细胞的发生进展中，由肿瘤细胞分泌的外泌体通过膜蛋白与靶细胞进行识别并融合，将其内部所含的miRNAs等物质释放至靶细胞，从而调节受体细胞的转录^[24]^。

外泌体利用分子机制和基因机制将其所携带的各类遗传信息以循环往复的方式进行近距离或远距离的传递^[36]^，参与细胞间的物质交换和信息交流活动，同时参与各种生理、病理过程和肿瘤细胞的生长及转移过程^[34]^。研究发现，由于外泌体在肺癌患者体内呈现异常表达的状态^[37]^，这提示着外泌体具有成为肺癌早期诊断生物标志物的巨大潜力^[38]^。外泌体作为一种细胞间通讯信息交换的载体^[39]^，目前在国内外已开展了外泌体细胞间信号转导、肿瘤诊断及靶向治疗等多领域的综合研究。

### 2.3 外泌体的提取与分离方法

外泌体作为一种生物信息载体，其实际价值如同血浆、胸水等，但其提取分离的特异性及效率却是一个影响其应用的重要问题。由于外泌体纳米级别的分子尺寸大小，外泌体的提取与分离方法一直以来是一个尚未彻底解决的问题。外泌体的异质性非常强，其颗粒直径大小不同，差异甚大，目前对于外泌体的分离和鉴定方法并无统一标准。由于缺乏特异性的标志物，目前暂时无法对提取效果做出客观判断。来自于肿瘤的生物标志物(外泌体)在血液样本中的表达丰度非常的低^[40]^，当前传统的外泌体提取和检测技术还远远不能满足高灵敏度提取和检测液体活检所需生物样品的要求。目前外泌体的提取方法有：超速离心沉淀法^[41]^、试剂盒提取法(Exo Quick^TM^法、qEV技术)^[42, 43]^、磁珠免疫分析法^[44]^及微流控技术等。这些技术在提取分析液体活检生物样品外泌体时都存在各自的优点与不足之处：提取费时费力、提取效率较低^[45]^、对外泌体损伤较大^[46]^、提取的外泌体容易受到蛋白的污染且提取纯度较低^[47]^等; 此外，试剂盒提取法(ExoQuick^TM^法、qEV技术)所运用到的试剂盒价格昂贵、所需成本较高^[48, 49]^，很难在临床和实际操作上实现大规模的运用。下面将具体阐述现有的各种提取外泌体的技术和方法。

#### 2.3.1 超速离心沉淀法

基于超速离心的外泌体分离技术被认为是外泌体提取与分离的金标准^[46]^，绝大部分外泌体研究中所使用的外泌体分离方法都是超速离心法。当非均质混合物(悬浮液)受到离心力作用时，悬浮液中的颗粒成分将根据其密度、大小和形状，将更大密度的或更大的颗粒首先沉淀出来^[45]^。离心通常用于分离和纯化颗粒材料以及分析聚合物材料(包括核酸和蛋白质等生物聚合物)的水动力特性。应用时，悬浮液中的颗粒可根据其物理性质和溶剂的密度和黏度依次分离^[41]^。在外泌体的分离方面，制备性超速离心是一个重要的部分，因为它的目的是分离病毒、细菌、亚细胞器和细胞外囊泡等小的生物制品^[45]^。

超速离心的方案一般开始时要使用亚微米过滤器或低速离心机去除杂质，如细胞碎片、微泡或凋亡小体。然后，使用超速离心机以至少100, 000×*g*的转速进行多次超速离心使外泌体沉淀，离心管管底产生的沉淀即外泌体^[46]^。除去上清液，然后将其重新悬浮在相对少量(200 μL左右)的PBS缓冲液中，产生浓缩样品，即可提取到外泌体。这种超高速的离心方法不仅需要大量的初始资金成本(超级离心机价格> 10万美元)，而且还需要大量的维护和运营成本; 除了高昂的费用之外，超速离心是一个耗时且劳动密集的过程，通常需要熟练技术人员或研究人员进行4 h-6 h的长时间工作^[41]^。为了满足实验所需的外泌体浓度和体积，还需要收集大体积(> 100 mL)的样品量^[50]^。最后，它仍然不会产生非常纯的样品，纯度只有5%-23%^[51]^。最终的外泌体样品仍然会受到蛋白质的污染，并且结果往往是高度可变的。

#### 2.3.2 磁珠免疫分析法

超速离心沉淀法的替代方案是通过磁珠和抗体功能化的支柱和填料进行免疫亲和捕获。外泌体的表面表达有大量的标志蛋白和特异性受体^[44]^，这为外泌体的高特异性提取提供了一个极好的机会。这些蛋白与其抗体可以发生免疫亲和识别作用，受体与其所对应的配体之间存在特异性结合作用^[44]^，因此，基于免疫亲和力捕获的外泌体分离技术已经被开发出来，这种磁珠免疫分析法适用于从其他流体组分中分离出外泌体。然而，该技术仅限于提取表面具有已知抗原的外泌体^[45]^。此外，由细胞分泌的大部分外泌体往往具有异源性，这大大限制了该方法的使用和推广。研究^[52]^表明，在不同来源的外泌体表面上能够大量表达的相同蛋白质并不常见。因此，使用基于磁珠免疫分析法的外泌体的提取方法目前仅成功地分离了存在于患者体内的一小部分外泌体。如果想要捕获所有的外泌体，而不仅仅是具有特定抗原的外泌体，磁珠免疫分析的方法是不充分不完全的。尽管磁珠加速了外泌体的后续分析，但购买磁珠所需成本较高且外泌体与磁珠的分离过程非常耗时，可能需要超过一天才能达到最佳回收率^[53]^。

#### 2.3.3 试剂盒提取法System Biosciences(SBI)

在2009年底发明了一种利用聚合物沉淀法进行外泌体分离的技术^[26]^。这项技术的工作原理是在“聚合物网”中捕获和收集一定尺寸范围(60 nm-150 nm)的外泌体，这些外泌体可以通过在离心机进行1, 500×*g*简单的低速离心进行回收^[46]^。目前该项技术已经用于商用，SBI将该项技术的商标名命名为Exo Quick^TM^和Exo Quick TC^TM^^[54]^。Exo Quick技术中的聚合物配方在特定的化合物条件下引入时形成网络，并且在4 oC-5 oC的条件下进行孵育^[55]^。外泌体作为单个纳米颗粒，需要通过超速离心获得很高的重力才能使外泌体颗粒脱离溶液，将它们聚集在由聚合物分支形成的聚合物袋或网中，这些细胞外纳米囊泡能够在低重力离心力的条件下恢复^[26]^。利用该种方法可提取获得外泌体颗粒，除去含有过量聚合物的上清液，将外泌体重新悬浮在合适的缓冲溶液中，如PBS溶液^[56]^。这个重悬过程稀释了外泌体颗粒中的残留聚合物，PBS溶解聚合物网并释放出完整的外泌体。Exo Quick^TM^法提取与分离外泌体的整个过程只需30 min^[57]^，这种方法所提取的外泌体产量高于超速离心沉淀法和磁珠免疫分析法。任何物种的生物流体样本中的外泌体都可以使用这种Exo Quick^TM^的技术进行分离^[57]^。但利用这种技术提取分离外泌体的方法所需的试剂盒价格昂贵、成本较高，目前很难在临床和实际操作中实现大规模应用。

近年来出现一种新型外泌体提取试剂盒，由IZON公司(新西兰)研发生产且已商业化使用的qEV试剂盒是一种根据尺寸排阻原理(size-exclusion chromatography, SEC)设计的外泌体提取分离技术^[58]^。qEV试剂盒可以从细胞上清液或各种生物样品中(血浆、血清、尿液、唾液等)高效、简便、无损地提取出外泌体，且提取的外泌体不包括囊泡聚合物，受蛋白污染的可能性较小，外泌体的生物活性较高^[59]^。但使用该技术每次提取的外泌体体积约为1.5 mL左右^[58]^，导致外泌体的浓度较低，如需提取高浓度的外泌体，则要进行进一步的外泌体富集与纯化工作。

#### 2.3.4 微流控技术

近年来利用微流控技术提取外泌体也较为广泛，Liu等^[60]^在2017年报道了一种基于尺寸分离的外泌体总分离芯片(exosome total isolation chip, ExoTIC)用于外泌体的提取与分离，该方法操作简便、易用、高通量、可自动化，利用微流控技术可从各种生物流体中利用压力驱动流体的方法高效分离出高纯度的外泌体。ExoTIC提取外泌体的产量比超速离心法高出4倍-1, 000倍，ExoTIC是一个模块化平台，它可以根据肿瘤细胞外泌体的大小对异源群体进行分类。Liu等^[60]^利用ExoTIC从癌症患者的临床生物样本中分离出外泌体，包括血浆、尿液和灌洗液，证明该装置对癌症和其他疾病具有广泛的适用性。该技术提取外泌体的关键核心为不同系列尺寸大小的纳米孔薄膜，由于该纳米孔薄膜的孔形为商业柱形孔，柱形孔在较强压力的驱动作用下承压能力较弱，易发生纳米孔形变，出现堵孔现象。因为该纳米孔薄膜的损伤不可逆，如果将该技术应用于大量生物样本外泌体的提取，需进一步优化纳米孔薄膜的承压能力，提高其广泛性和实用性。

## 3 外泌体在肺癌早期诊断中的作用

由肿瘤分泌的外泌体几乎可以在所有类型的体液中有效检测到。由于外泌体成分极其复杂，含有多种蛋白、核酸等，不同疾病、不同个体甚至不同病期都有很大差异。外泌体内部所含的miRNA、蛋白质等生物分子的稳定性较高，相比于游离miRNA和蛋白质更加稳定，可如实地反映其分泌细胞的生理状态及病理特征，作为液体活检的检测标志物，外泌体具有良好的生物学前景。

### 3.1 外泌体的蛋白标志物

外泌体中含有多种蛋白质成分，如表面蛋白与胞内蛋白，可以促进肺癌的发生发展，与肺癌的早期诊断与预后有着密切的联系^[61]^。大量外泌体的标志性膜蛋白可以作为肺癌的诊断生物标志物，如目前已发现的有CD91、CD317、表皮生长因子受体(epidermal growth factor receptor, EGFR)等^[62]^。研究^[63]^表明，用生物素结合的CD151、CD171和tetraspanin8抗体的混合物来检测和捕获外泌体比传统的外泌体提取方法有着更强的外泌体分离能力。更重要的是，研究发现外泌体中CD151、CD171、tetraspanin8的水平在肺癌患者中高表达，其表达水平明显高于正常人，进而提示着我们CD151、CD171、tetraspanin8有着作为强有力的肺癌早期诊断的蛋白标志物的潜力。另外，NY-ESO-1、EGFR、PLAP、上皮细胞黏附分子(epithelial cell adhesion molecule, EpCAM)和Alix等外泌体膜表面蛋白对预测肺癌患者远期总体生存率有显著的作用，可以作为潜在的预后标志物^[34]^。

除了外泌体的表面标志性膜蛋白外，实验人员发现外泌体中所含有的蛋白质也可作为肺癌早期诊断的生物标志物。David等^[64]^用氨基酸稳定同位素定量标记(stable isotope labeling with amino acids, SILAC)的定量蛋白组学方法分析了721种外泌体蛋白，并发现了许多与增殖相关的细胞信号分子，如SRC、EGFR等与信号转导相关的蛋白在NSCLC外泌体中富集，并能积极调节肿瘤受体细胞增殖。通过对NSCLC外泌体蛋白组的研究，有助于发现与肺癌进展相关的外泌体富集蛋白物质，这可能对NSCLC生物标志物的发现和发展具有潜在的临床意义。Niu等^[65]^研究了125例NSCLC患者和46名正常人血清中肿瘤衍生的外泌体生物标志物，以提高中国NSCLC患者的诊断价值。与健康对照组相比，NSCLC患者外泌体中AHSG和ECM1的表达水平显著升高，表明在NSCLC患者血清外泌体中发现的蛋白标志物AHSG和ECM1显示出潜在的诊断价值。

### 3.2 外泌体的核酸标志物

外泌体中的RNA是其内含物中重要的组成部分，肺癌患者外泌体中的RNA较正常人群表达水平明显升高，这与肺癌的发生发展、侵袭转移等生物学特性密切相关^[66]^。近年来有大量文献报道，外泌体中的循环miRNAs可作为肺癌的潜在诊断标志物。Cazzoli等^[67]^从30名受试者的血浆外泌体中鉴定出4种miRNA(miR-378a、miR-379、miR-139-5p和miR-200b-5p)，以筛选和区分肺癌患者和健康对照组。与外泌体蛋白类似，外泌体miRNA的表达水平也可以作为评估肺癌患者预后的指标。Liu等^[68]^的研究结果表明，高表达水平的外泌体miRNA(miR-23b-3p、miR-10b-5p、miR-21-5p)与肺癌患者的不良预后明显相关，并且联合多种miRNA分析具有更高的敏感性与特异性。Cecilia等^[22]^研究发现miR-21的表达增加与NSCLC患者的预后恶化有关。有研究^[69]^发现在NSCLC来源的外泌体的miRNA中，miR-10b-5p、miR-15b-5p具有诊断鳞癌的特异性，而miR-181-5p、miR-30a-3p、miR-30e-3p和miR-361-5p具有诊断腺癌的特异性，通过下一代测序(next-generation sequencing, NGS)平台检测miR-320b，验证了其诊断的准确性。

根据Zhang等^[70]^的报道，肺癌患者术后外泌体中miR-500a-3p、miR-501-3p和miR-502-3p的表达显著上调，这提示着miR-500a-3p、miR-501-3p和miR-502-3p可能与肿瘤的进展有关，这些变化也可能与肿瘤生长过程中持续的炎症反应相关。分析显示miR-500a-3p、miR-501-3p和miR-502-3p的上调与肺癌患者总体生存率的提高有关，虽然这三种miRNAs在肺癌中的发生机制尚不清楚，但在其他癌症类型中也观察到它们的促肿瘤作用。这提示着我们，外泌体中miR-500a-3p、miR-501-3p和miR-502-3p有着作为肺癌早期诊断标志物的巨大潜力。此外，苏超粤等^[71]^调研文献发现，利用miRNA测序和定量聚合酶链反应(q-polymerase chain reaction, qPCR)技术检测41例Ι期NSCLC患者与42名健康正常人中的血浆外泌体miRNA及其Ct值，以筛选和确定NSCLC特异性的miRNA，结果发现miR-181b-5p、miR-361b-5p、miR-10b-5p和miR-320b这4种miRNA具有鉴别肺腺癌、肺鳞癌及非NSCLC患者的能力。因此，通过非侵入性的方式(液体活检)检测外泌体中具有差异表达的miRNA可用于肺癌的早期诊断。鉴于以上优点，外泌体有望替代传统的肺癌筛查工具及检查方法应用于临床工作中，为肺癌的早期诊断提供新的技术思考并奠定坚实的理论依据。

## 4 展望

外泌体在肺癌的发生发展过程中具有重要作用。外泌体作为肿瘤微环境中细胞间交流的载体，促进了肺癌的发生发展过程。同时，它也是肺癌诊断及预后的潜在生物标志物。要探索外泌体在肺癌的早期诊断中所发挥的作用，首先要建立一套外泌体专属的提取与分离方法，而当前传统的外泌体提取和分离技术还远远不能满足高灵敏度提取和分离液体活检所需外泌体的要求。这需要我们研发出更好的外泌体提取与分离方法，进一步推动外泌体与肺癌早期诊断研究的发展，这具有重要的基础研究价值和潜在应用价值。同时，加速外泌体生物标志物的发现、验证、监管批准，最终快速运用到临床，为临床更加科学的进行肺癌早期诊断、分型分期、精准治疗和预后判断提供新思路和新途径。
